# Intranasal immunization with recombinant Lactococci carrying human papillomavirus E7 protein and mouse interleukin-12 DNA induces E7-specific antitumor effects in C57BL/6 mice

**DOI:** 10.3892/ol.2013.1743

**Published:** 2013-12-09

**Authors:** YIJIE LI, XINPING LI, HUANHUAN LIU, SHUZHEN ZHUANG, JIANHUA YANG, FUCHUN ZHANG

**Affiliations:** 1 Xinjiang Key Laboratory of Biological Resources and Genetic Engineering, College of Life Science and Technology, Xinjiang University, Urumqi, Xinjiang 830046, P.R. China; 2 Department of Pediatrics, Texas Children's Cancer Center, Dan L. Duncan Cancer Center, Baylor College of Medicine, Houston, TX 77030, USA

**Keywords:** *Lactococcus lactis*, co-delivery, intranasal immunization, human papillomavirus, antitumor

## Abstract

The use of *Lactococcus lactis* for the co-delivery of antigens and cytokines has been shown to successfully induce a special immune response. However, it is unknown whether the same results may be triggered through immunization of animals with *L. lactis* simultaneously carrying protein antigen and cytokine DNA. The present study evaluated the protective effects of intranasally administered live *L. lactis* strains carrying human papillomavirus 16 E7 protein and murine interleukin-12 (IL-12) DNA (LL-E7_P_-IL-12_D_) in a TC-1 tumor animal model. C57BL/6 mice were intranasally immunized with recombinant lactococci, and assays for cytotoxicity measurement and tumor protection were carried out to assess the immunological effects of the vaccine candidates. IL-12 and interferon-γ serum levels were measured and immunization with LL-E7_P_-IL-12_D_ was shown to induce an E7-specific immune response and to confer protection against TC-1-induced tumors *in vivo*. Mice in the LL-E7P-IL-12D group showed an 80% survival rate when the control mice had died. Therapeutic immunization with recombinant *L. lactis* strains 7 days after TC-1 injection led to a reduction in the number of palpable tumors in treated mice. The antitumor effects of the vaccination occurred through an E7-specific cytotoxic T-lymphocyte response. In the present study, the use of a single *L. lactis* strain, to co-administer protein antigen and adjuvant DNA, successfully induced an antigen-specific immune response. These observations demonstrate a new strategy for the use of *L. lactis* as a delivery vector of therapeutic molecules and antigens.

## Introduction

Vaccines and protein therapeutics are most commonly administered by injection. However, the use of this method presents a number of limitations for large vaccination programs, particularly in developing countries. Such limitations include costs, the need for trained persons and the stress associated with immunization in adults and children (1). Mucosal immunization is another recognized means of administering vaccine antigens to humans and is one of the few needle-free immunization methods commercially available or under development. This method involves the delivery of vaccines to a mucosal membrane, for example the ocular, oral, nasal, pulmonary, vaginal or rectal membranes. Mucosally administered vaccines have been shown to stimulate serum IgG and mucosal IgA antibodies, as well as induce cytotoxic T-lymphocyte (CTL) activities (2). The administration of therapeutic molecules via mucosal routes may present an efficient prophylactic and therapeutic strategy given that mucosal surfaces are the major site of entry for pathogens. While injected vaccinations are generally weak or poor inducers of mucosal immunity, the administration of vaccines onto mucosal surfaces presents a highly efficient method for inducing mucosal immune responses (3). This activity is critical for immune protection as local mucosal immune responses are important for protecting the mucous membranes against infection by pathogenic microorganisms.

Over the last two decades, a number of studies have focused on the use of *Lactococcus lactis* as a mucosal delivery vector for therapeutic proteins and antigens (4–6). The available data demonstrate that *L. lactis* is an excellent tool for the controlled and targeted administration of vaccine antigens to the mucosal immune system. One major advantage of *L. lactis* as a delivery vehicle is that this food-grade dairy microorganism is generally regarded as safe and has been widely consumed by humans in fermented foods for centuries. *L. lactis* is noninvasive, nonpathogenic, non-commensal and does not colonize normal tissues. The second major advantage of this bacterium as a mucosal delivery vehicle is that, in addition to its efficient elicitation of antigen-specific mucosal immune responses, it also reduces the potential side effects common to systemic routes of administration. The immune response elicited against the *L. lactis* vector itself is only a weak one, while the major immune responses are directed primarily against the heterologously expressed antigens (5,7). Therefore, the possibility of a strong immune response against the vaccine carrier, diminishing the response against the heterologous antigens, is avoided. Additionally, restrictive time limits for usage due to anti-vector immunological responses are also avoided. A third advantage of *L. lactis* as a delivery vehicle is that it may be engineered to simultaneously express multiple proteins and other molecules, including antigens and adjuvants, multivalent protective antigenic determinants and various suicide genes. The simultaneous expression of multiple foreign genes in a single *L. lactis* strain affects the extent to which a given gene may be expressed. However, these negative effects may be avoided if these genes are expressed in prokaryotic and eukaryotic systems.

Human papillomavirus (HPV) is a double-stranded DNA tumor virus specific to squamous epithelial cells of the skin and mucous membranes. Persistent infections arising in those with high-risk genotypes of HPV have been causally linked to the incidence of cervical cancer. An HPV prophylactic vaccine has been successfully developed and has received approval for its use worldwide. However, while prophylactic vaccines composed of L1 virus-like particles are available and have been shown to prevent HPV infection with the virus types contained in the vaccine (8), they are unable to treat the millions of patients who are already infected (9). HPV E7 oncogenic protein is an ideal tumor-specific antigen for HPV therapeutic vaccines as it is present only in tumor cells, is essential in cellular transformation and is constitutively expressed in HPV-associated malignancies and their precursor lesions (10). As HPV-16 is the most prevalent example of the high-risk HPV genotypes, several HPV E7 protein systemic and mucosal vaccines have been assessed for their ability to elicit an immune response against HPV-16 (11–15). As with other cancer antigens, adjuvants are necessary to enhance the desired immune response to E7 protein. Among the cytokines tested as molecular adjuvants, interleukin-12 (IL-12) has been recognized as the most effective for enhancing antigen-specific cellular responses in a number of vaccine model systems. Some studies have shown that significant antitumor immunity against TC-1 tumors may be induced by the co-delivery of IL-12 and E7 (11,12). The present study utilized cell-wall-weakened single recombinant lactococcal strains carrying HPV-16 E7 protein and the IL-12 gene for intranasal (i.n.) immunization in mice, distinct from the co-administration of one recombinant lactococcal strain carrying HPV-16 E7 protein and a second strain carrying the IL-12 gene. The antitumor effects observed were compared with those from previous studies.

## Materials and methods

### Cell line strains in mice

The TC-1 lung tumor cell line was produced for use in mice by transduction with a retroviral vector expressing HPV-16 E6-E7 combined with a retrovirus expressing activated c-Ha-ras (16). TC-1 cells were grown in RPMI-1640 supplemented with 10% fetal calf serum, 50 U/ml penicillin, 50 U/ml streptomycin and 0.4 mg/ml G418. B16 cells were kept in the laboratory and cultured in Dulbecco's modified Eagle's medium-10% fetal bovine serum (FBS) at 37°C in 5% CO_2_.

Female C57BL/6 mice aged between 6 and 8 weeks were used for these studies. The animals were purchased from the Institute of Genetics of the Chinese Academy of Sciences and used in accordance with the animal protocols approved by the Institutional Research Committee.

### Preparation of live L. lactis vaccines

*L. lactis* NZ3900 and the *E. coli*-*L. lactis* shuttle vector pMG36e plasmid were purchased from MoBiTec GmbH (Göttingen, Germany) and Yrgene (Changsha, Hunan, China), respectively. *L. lactis* was grown in M17 (Difco Laboratories, Inc., Franklin Lakes, NJ, USA) supplemented with 0.5% glucose at 30°C without shaking. For construction of LL-E7_P_-IL-12_D_, the full protein-coding region of the HPV-16 E7 gene was amplified by polymerase chain reaction (PCR) from vector pcDNA3-E7_M_(17) using the following primer pair: Sense primer, 5′-GTTgagctcATGGAGATACACCTA CATTGC-3′ and antisense primer, 5′-GCCtctagaATG GTTTCTGAGAACAGATGG-3′. The amplified PCR product was cloned into the *Sac*I-*Xba*I site of pMG36e, resulting in formation of the plasmid pMG36e-E7_P_, in which the E7 gene is under control of the P32 promoter in the sense orientation. The eukaryotic expression cassette with the cDNA of mouse IL-12 was amplified by PCR from the pOFR-mIL-12 vector (InvivoGen, San Diego, CA, USA) using the following primer pair: Sense primer, 5′-TGTCTAAAAAGCTAGCTCG AGCGGCCGCAAT-3′ and antisense primer, 5′-TGTCTAAAA AGCTAGCGATCTACCACATTTGTAGAGG-3′. The PCR product was subcloned into the *Nhe*I sites of pMG36e-E7_P_ and pMG36e using in-fusion technology, resulting in the formation of plasmids pMG36e-E7_P_-IL-12_D_ and pMG36e-IL-12_D_. The plasmids were then transformed into *E. coli* DH5α or *L. lactis*. Transformation of *L. lactis* NZ3900 was performed as described previously (18), with certain modifications. Briefly, a lactococcal culture was grown in 5 ml GM17 broth overnight at 30°C. On the second morning, the culture was inoculated into 20 ml pre-warmed GM17 broth, followed by incubation at 30°C for 2–3 h to reach the early exponential phase (OD_600_, 0.3–0.6). Penicillin G was added to a final concentration of 100 μg/ml and the culture was incubated for 1 h. The cells were harvested by centrifugation, resuspended in 1 ml lithium acetate solution [100 mM LiAc, 10 mM DTT, 0.6 M sucrose and 10 mM Tris-HCl (pH 7.5); filter-sterilized] and incubated for 30 min at room temperature. After washing the cells twice with sterile deionized water, once with 50 mM EDTA and three times with sterile deionized water, the cells were resuspended in 0.2 ml sucrose (0.3 M). Competent cells were added to the ligation mixture and this was treated using a Gene Pulser apparatus (Bio-Rad, Richmond, CA, USA) according to the manufacturer's instructions. The electroporated mixture was immediately diluted with 1 ml GM17 broth containing 20 mM MgCl_2_ and 2 mM CaCl_2_ prior to incubation for 2 h at 30°C. The mixture was subsequently plated onto M17 plates containing 0.5 M sucrose and 10 μg/ml erythromycin for the selection of pMG36e-E7_P_, pMG36e- IL-12_D_ and pMG36e-E7_P_-IL-12_D_.

The selected LL-E7_P_, LL-IL-12_D_ and LL-E7_P_-IL-12_D_ strains were grown at 30°C in GM17 supplemented with 10 μg/ml erythromycin. For the cell-wall-weakening treatment, the overnight cultures were diluted at a ratio of 1:5 (2 ml into 8 ml) with pre-warmed G-SGM17 medium (0.5% w/v glucose, 0.5 M sucrose and 2.5% w/v glycine). Following incubation of the cultures at 30°C for 2 h, the cells were harvested, washed three times with sterile 10% glycerol and resuspended in phosphate-buffered saline (PBS) at a final concentration of 1×10^9^ colony-forming units (CFU).

### Western blot analysis

E7 and IL-12 protein samples were separated on a 12% SDS polyacrylamide gel. The separated proteins were electrophoretically transferred to a nitrocellulose membrane (GE Healthcare Bio-Sciences, Pittsburgh, PA, USA). Subsequent procedures were performed according to the manufacturer's instructions for the Chromogenic Western Blot Immunodetection kit (Invitrogen Life Technologies, Carlsbad, CA, USA).

### Tumor protection assay

Female C57BL/6 mice aged 8–10 weeks were used to evaluate protective effects against the experimental tumor. Groups of mice were immunized intranasally with 1×10^9^ CFU of each recombinant *L. lactis* strain: LL-E7_P_ alone; LL-E7_P_ co-immunization with LL-IL-12_D_ (LL-E7_P_/IL-12_D_); or LL-IL-12_D_ and LL-E7_P_-IL-12_D_. The samples were suspended in 10 μl PBS and 5 μl was administered into each nostril on days 0, 14 and 28 using a micropipette. Control mice received identical quantities of an isogenic strain of *L. lactis* containing an *L. lactis* expression cassette with red fluorescent protein cDNA and a eukaryotic expression cassette with enhanced green fluorescent protein cDNA (LL-R_P_-G_D_) (19). Seven days after the final administration (day 35), the mice were challenged with subcutaneous injection of 5×10^4^ TC-1 tumor cells in 100 μl PBS in the right rear flank. The dimensions of the tumor at the site of the TC-1 cell injection were measured (two perpendicular measurements) every week using a caliper and the volume of the tumor was estimated using the following formula: Volume (cm^3^) = (length × width^2^)/2.

For the therapeutic experiments, the mice were first challenged subcutaneously with 5×10^4^ TC-1 tumor cells in the right rear flank. When palpable tumors (tumor mass, ≥5 mm in diameter) were observed in the mice, live recombinant lactococci were administered at three 7-day intervals (days 7, 14 and 21). At day 7, 100% of the challenged mice had developed a palpable tumor. Tumor growth was monitored weekly.

### CTL response against TC-1 tumor cells

In order to characterize the splenocytes obtained from the vaccinated mice, *in vitro* stimulation was performed and CTL activity was measured [Lactate Dehydrogenase (LDH) Cytotoxicity Detection kit, Roche Applied Science, Penzberg, Germany] based on the measurement of LDH release from lysed cells. Mouse splenocytes, obtained from each group (n=5), were harvested 7 days after the final immunization. The splenic cells were cultured in complete RPMI-1640 medium supplemented with 10% FBS, IL-2 (10 U/ml) and glutathione S transferase (GST)-E7 (10 μg/ml, expressed and purified using an *E. coli* expression system) for 4 days. TC-1 cells containing the HPV-16 E6/E7 gene and activated human c-Ha-ras oncogene were used as the target cells. A melanoma cell line (B16) derived from C57BL-6 mice served as a non-E7 expressing reference in this experiment. Target cells and effector cells were resuspended in an assay medium (RPMI-1640 with 1% bovine serum albumin) and the target cells (5×10^4^ cells) were co-cultured with the effector cells at a ratio of 12.5:1, 25:1 or 50:1 (tested in duplicate) in 96-well round-bottom culture plates at 37°C. After 4 h of incubation, the culture plates were centrifuged and the supernatants (100 μl per well) were transferred to a new ELISA plate. The LDH detection mixture was added (100 μl per well) and the samples were incubated in the dark for 30 min at room temperature. Following the addition of a stop solution (1 M H_2_SO_4_), the absorbance of the samples was measured using an ELISA plate reader (Bio-Rad) at 490 nm. The spontaneous release of LDH by target or effector cells was assessed by incubation of the target cells in the absence of the effector cells and vice versa. The maximum release of LDH was determined by incubating the target cells in an assay medium containing 1% NP-40. The percentage of specific cell-mediated cytotoxicity was calculated using the following equation: Specific cytotoxicity (%) = [(experimental value - spontaneous LDH release) - (maximum LDH release - spontaneous LDH release)] × 100.

### ELISA detection of E7-specific antibodies and measurement of IL-12 and interferon (IFN)-γ expression levels

Serum was collected from the orbital veins of tumor-bearing mice following the therapeutic injections. For the detection of IgG and IgA E7-specific antibodies, 96-well microtiter plates were coated with 10 μg/ml of the purified GST-E7 fusion protein diluted in 100 mM carbonate buffer [50 mM sodium carbonate and 1 mM MgCl_2_ (pH 9.8)] overnight at 4°C. The plates were washed twice with PBS-Tween (PBS containing 0.02% Tween) and blocked with 3% bovine serum albumin for 1 h at room temperature. The plates were then washed with PBS-Tween and the diluted samples were added to the wells (in triplicate). Following this, the plates were incubated for 2 h at 37°C. Following washing with PBST (PBS with 0.05% Tween-20), a goat anti-mouse IgG or IgA horseradish peroxidase (Sigma, St. Louis, MO, USA) was added, followed by a further 2 h incubation at 37°C. The plates were washed and developed with TMB (Sigma) for 10–20 min at room temperature. The reaction was stopped by adding 2 M NaOH and the absorbance was immediately measured at 450 nm. IL-12 and IFN-γ levels were determined using IL-12p70 and IFN-γ ELISA kits (Wuhan Boster Biological Technology, Ltd., Hubei, China), respectively, according to the manufacturer's instructions.

### Statistical analyses

The results are presented as the mean ± standard error of the mean. Student's t-test was used for data analysis and P<0.05 was considered to indicate a statistically significant difference.

## Results

### Expression of E7 and IL-12 in vitro

**Figure 1:**
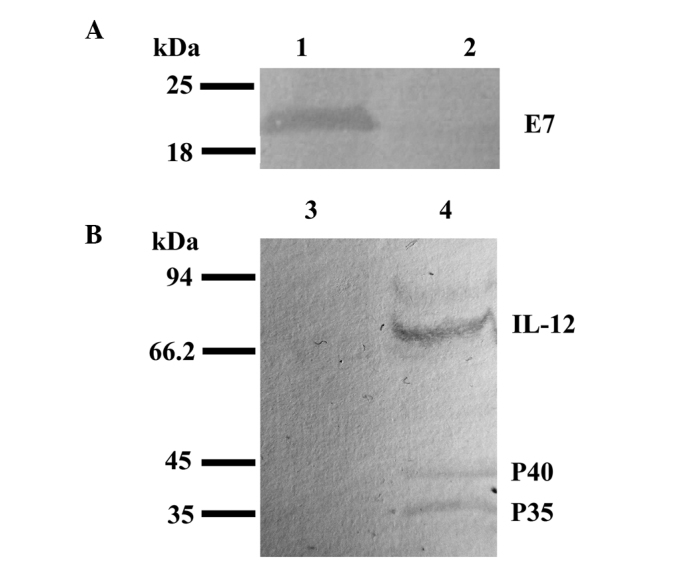
Western blot analysis of recombinant protein expressed in *Lactococcus lactis* or 293T cells. (A) Production of human papillomavirus-16 E7 by recombinant *L. lactis* was analyzed using anti-E7 antibodies. E7 antigen was detected as a 20-kDa band in lane 1. (B) IL-12 production was analyzed by immunoblotting using anti-IL-12 antibodies following transfection of 293T cells with recombinant *L. lactis* (LL-IL-12D). IL, interleukin.

To confirm E7 protein expression in the *L. Lactis* strains, E7 protein levels were detected by western blot analysis (Fig. 1A). These data show that E7 protein was expressed in LL-E7 (lane 1) but not in LL-R_P_-G_D_ (lane 2). The E7 protein was detected as an ~20-kDa band in lane 1.

In order to confirm IL-12 protein expression in the LL-IL-12_D_-transfected 293T cells, IL-12 protein levels were detected by western blot analysis (Fig. 1B). These data show that IL-12 protein was expressed in LL-IL-12_D_-transfected cells (lane 4) but not in LL-R_P_-G_D_-transfected cells (lane 3). The IL-12 protein was detected as an ~70-kDa band in lane 4.

### Immunogenicity assay in mice

**Figure 2:**
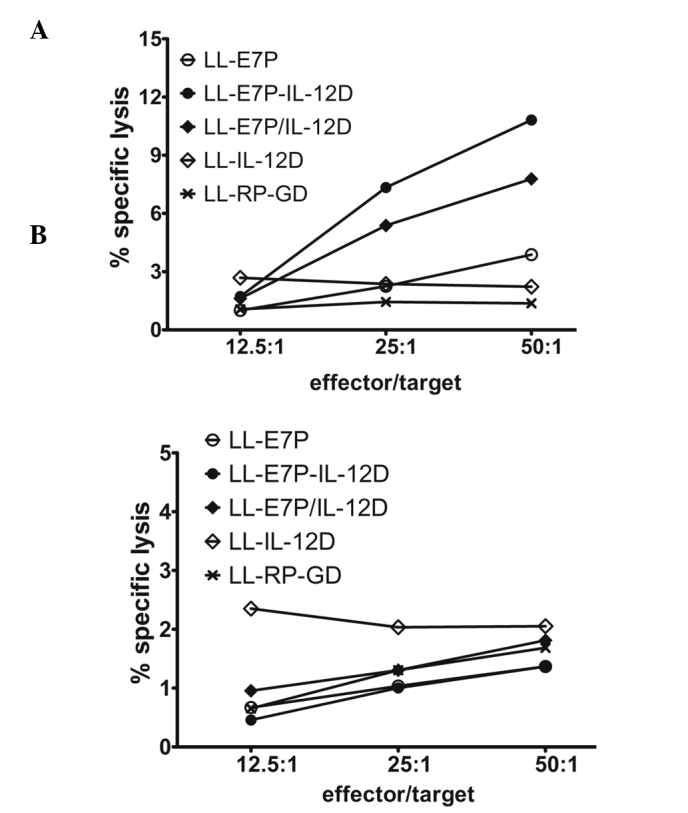
Induction of an E7-specific CTL response in the splenocytes of mice vaccinated with recombinant lactococci. Splenocytes from vaccinated mice were pooled 7 days after the final injection and treated with glutathione S transferase-E7 *in vitro*. A lactate dehydrogenase assay was used to assess CTL lytic activity with (A) TC-1 or (B) B16 cells as the target cells. CTL, cytotoxic T-lymphocyte; IL, interleukin.

Antibody titer analysis and a CTL assay were carried out and a limited antibody response was induced by the LL-E7_P_-IL-12_D_ and LL-E7_P_/IL-12_D_ vaccine candidates. In order to characterize the splenocytes from the immunized mice, *in vitro* stimulation and measurement of CTL activity were performed. As shown in Fig. 2A, immunization with LL-E7_P_-IL-12_D_ or LL-E7_P_/IL-12_D_ elicited significant E7-specific CTL activity *in vivo*. However, animals immunized with LL-R_P_-G_D_, LL-IL-12_D_ or LL-E7_P_ alone exhibited minor CTL activity. These results indicate that mucosal vaccination with live LL-E7_P_-IL-12_D_ or LL-E7_P_/IL12_D_ induces E7-specific CTL cells, which are likely to be responsible for tumor protection. Furthermore, the CTL responses to B16 cells in the five mouse groups were low (Fig. 2B). These results indicate that live LL-E7_P_-IL-12_D_ and LL-E7_P_/IL12_D_ vaccine candidates have the potential to induce CTL to kill target cells containing E7 oncogenes.

### Tumor protection mediated by immunization with LL vaccine candidates

**Figure 3:**
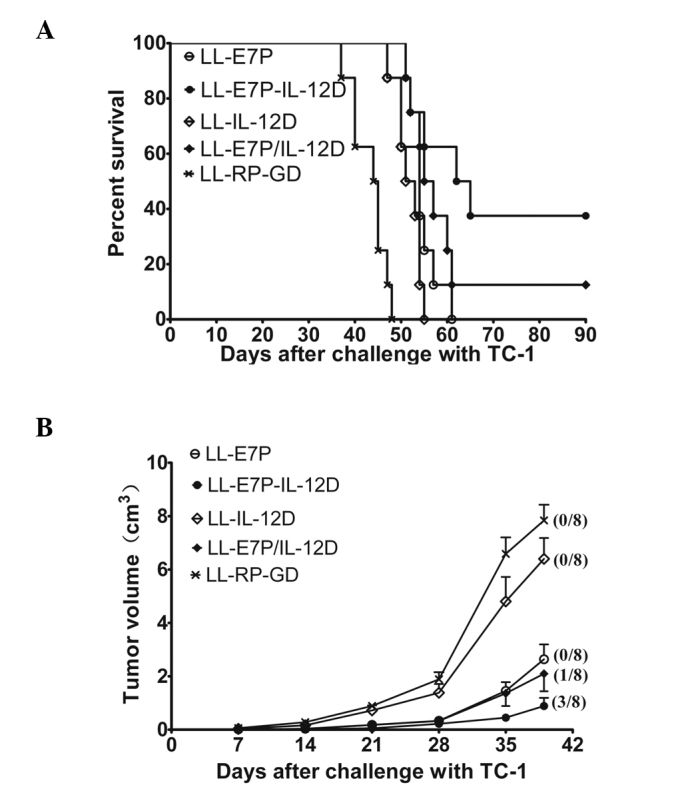
*In vivo* tumor protection experiments with recombinant lactococci. Mice (n=8) were immunized on days 0, 14 and 28 with LL-E7P, LL-IL-12D, LL-E7P-IL-12D, LL-E7P/IL-12D or LL-RP-GD and a challenge using the TC-1 tumor cell line was performed (day 35). The data indicate (A) survival rates and (B) tumor mass progression (the numbers in parentheses indicate the proportion of tumor-free animals) monitored at 7-day intervals. The tumors of mice immunized with LL-E7 were significantly smaller than those of mice immunized with LL-IL-12D (P<0.0001) and LL-RP-GD (P<0.0001). The tumors of mice treated with LL-E7P-IL-12D were significantly smaller than those of mice immunized with LL-E7, LL-IL-12D and LL-RP-GD (P<0.05, P<0.001 and P<0.0001, respectively). The tumors of mice treated with LL-E7P/IL-12D were significantly smaller than those of mice immunized with LL-IL-12D (P<0.0001) and LL-RP-GD (P<0.0001), but there was no significant difference in tumor size between mice treated with LL-E7P and mice treated with LL-E7P-IL-12D. IL, interleukin.

To determine whether i.n. administration with LL-E7_P_-IL-12_D_ produced antitumor effects, an *in vivo* tumor protection experiment was performed using an HPV-16 tumor model in which injection of the TC-1 cell line promotes the formation of lethal tumors. The protective efficacy of LL-E7_P_-IL-12_D_ was first analyzed when used as a prophylactic vaccine. The groups of mice were immunized intranasally three times (at 2-week intervals) with LL-E7_P_, LL-IL-12_D_, LL-E7_P_/IL-12_D_ or LL-E7_P_-IL-12_D_. Seven days after the final immunization, the mice were challenged subcutaneously with 5×10^4^ TC-1 and monitored once a week. As a negative control, a group of mice were administered with an isogenic strain of *L. lactis* (LL-R_P_-G_D_) that produces proteins unrelated to E7 and IL-12. As shown in Fig. 3, 100% of the mice vaccinated with LL-R_P_-G_D_ or LL-IL-12_D_ alone developed aggressive tumors that led to mortality within 55 days (Fig. 3A). The median tumor sizes in LL-R_P_-G_D_ and LL-IL-12_D_ groups after 35 days were ~7.84 and ~6.39 cm^3^, respectively (Fig. 3B). The majority of mice treated with LL-E7_P_ or LL-E7_P_/IL-12_D_ developed aggressive tumors 3 weeks after TC-1 administration and the median tumor sizes at 35 days (~2.64 and P<0.0001; ~2.1cm^3^ and P<0.0001, respectively) were 2-fold lower than those of the LL-R_P_-G_D_ and LL-IL-12_D_ mice at 35 days. Administration of LL-E7_P_-IL-12_D_ yielded the most effective response: 37.5% of the mice remained tumor-free for the duration of the test period (~90 days). For the remaining 62.5% of tumor-bearing mice, the median tumor size (~0.89 cm^3^) was significantly reduced compared with the LL-R_P_-G_D_ (P<0.0001), LL-IL-12_D_ (P<0.0001) and LL-E7_P_ (P<0.05) mice. Additionally, the antitumor effect elicited by LL-E7_P_-IL-12_D_ immunization appeared to be long-lasting: Three tumor-free animals were re-challenged 3 months later with TC-1 cells in the opposite flank and remained tumor free for up to 6 months.

**Figure 4:**
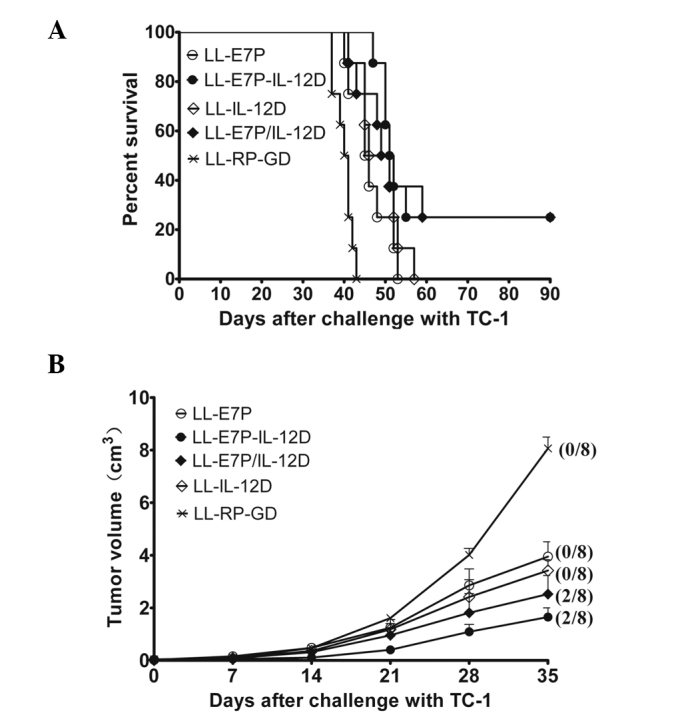
Immunotherapy with recombinant lactococci. Each group of mice (n=8) was inoculated subcutaneously with 5×10^4^ TC-1 cells immunized with LL-E7P, LL-IL-12D, LL-E7P-IL-12D, LL-E7P/IL-12D or LL-RP-GD on days 7, 14 and 21. The data indicate (A) survival rates and (B) tumor mass progression (the numbers in parentheses indicate the proportion of tumor-free animals) monitored at 7-day intervals. The tumors of mice treated with LL-E7P or LL-IL-12D were significantly smaller than those of mice treated with LL-RP-GD (P<0.0001 and P<0.0001, respectively) and the tumors of mice treated with LL-E7P-IL-12D were significantly smaller than those of mice immunized with LL-E7P, LL-IL-12D and LL-RP-GD (P<0.005, P<0.005 and P<0.0001, respectively). The tumors of mice treated with LL-E7P/IL-12D were significantly smaller than those of mice immunized with LL-RP-GD (P<0.0001), but there were no significant differences in tumor size among mice treated with LL-E7P, LL-IL-12D and LL-E7P-IL-12D. IL, interleukin.

The therapeutic effects of administration with LL-E7_P_-IL-12_D_ were also evaluated in mice that had previously been injected with the TC-1 tumor cell line prior to the beginning of the immunotherapy protocol. As soon as a palpable tumor mass developed (≥5 mm in diameter), the immunotherapy treatment was initiated. As shown in Fig. 4, LL-E7_P_-IL-12_D_ and LL-E7_P_/IL-12_D_ treatments resulted in total tumor regression in 25% of the immunized animals, which remained tumor-free for the duration of the test period (~90 days). The LL-E7_P_ immunization group exhibited a 51% reduction in tumor size. The reductions in tumor size observed for LL-IL-12_D_ and LL-E7_P_/IL-12_D_ groups were ~58 and ~69%, respectively. The LL-E7_P_-IL-12_D_ group exhibited a significant decrease in growth of ~80% compared with the LL-R_P_-G_D_ immunization group.

Although LL-E7_P_ and LL-IL-12_D_ treatment did not induce total tumor regression, certain decreases were observed when comparing these groups with the LL-R_P_-G_D_ treatment group.

Tumor-free mice in the prophylactic and therapeutic experiments appeared to be healthy at the end of the experimental period and no weight loss was observed. Furthermore, no visible toxicity was detected during autopsy. Overall, these observations reveal that the administration of LL-E7_P_-IL-12_D_ induces antitumor effects against an HPV-16 tumor model in mice.

### IL-12 and IFN-γ expression levels in the serum

**Figure 5:**
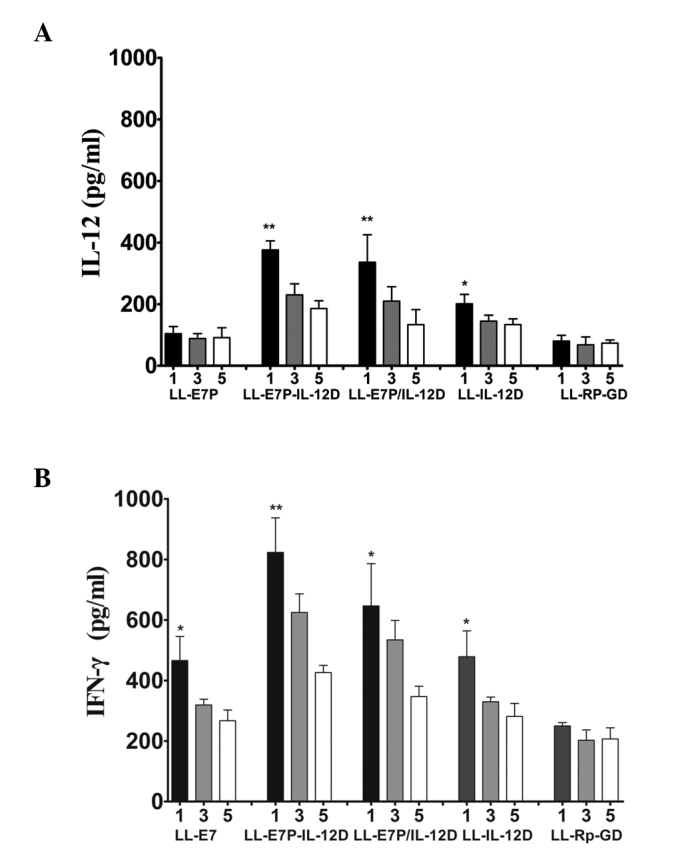
Systemic cytokine expression levels in the serum at various time points using a murine TC-1 tumor model. Mice were intranasally immunized with recombinant lactococci. At the indicated time points, (A) IL-12 and (B) IFN-γ levels in the serum were determined by ELISA. The results shown are representative of three independent experiments. ^*^P<0.05, vs. the LL-RP-GD group and ^**^P<0.01,vs. the LL-RP-GD group. IL, interleukin; IFN, interferon.

In addition to its use as a molecular adjuvant, IL-12 exhibits potent antitumor activity. IL-12-induced IFN-γ has been shown to upregulate major histocompatibility complexes and adhesion molecules, resulting in enhanced susceptibility of tumor cells to CTL-mediated killing. The levels of IL-12 and IFN-γ in the serum of a murine TC-1 tumor model were therefore measured in the present study. Time-dependent IL-12 and IFN-γ expression levels in the serum were measured by sandwich ELISA following i.n. immunization of the mice with recombinant lactococci. As shown in Fig. 5, LL-E7_P_-IL-12_D_ and LL-E7_P_/IL-12_D_ appeared to elicit higher and more sustained expression of IL-12 and IFN-γ in the serum compared with the other treatments. However, there was no marked difference between LL-E7_P_-IL-12_D_ and LL-E7_P_/IL-12_D_ groups. A subsequent reduction in IL-12 and IFN-γ serum expression levels was observed until day 5. In this study, LL-E7_P_ also elicited higher expression levels of IFN-γ in the serum compared with the LL-R_P_-G_D_ control but sustained expression was not observed. By contrast, mice immunized with LL-R_P_-G_D_ exhibited low expression levels of IL-12 and IFN-γ in the serum. These results indicate that enhanced and prolonged expression of cytokines is closely associated with the induction of strong tumor-specific T-cell responses and a subsequent increase in antitumor effects.

## Discussion

Research has shown that effective vaccines consist of a specific moiety, i.e., the structures that present the protective antigenic determinants, and a nonspecific moiety, i.e., the adjuvant components (20). With regard to cancer antigens, adjuvants are required to enhance the desired immune response to weak antigens (21). The *L. lactis* antigen delivery system works in this way. A number of studies focusing on the ability of *L. lactis* to secrete biologically active cytokines have shown that mucosal and systemic responses may be enhanced by the co-expression (and secretion) of cytokines and antigen (11,22). Recent studies have also shown that *L. lactis* may be used to deliver DNA molecules to mammalian cells as vaccines or as a form of gene therapy (18,23–25). In contrast to bacterial-mediated delivery of proteins expressed primarily in their denatured form, bacterial-mediated delivery of eukaryotic genes may facilitate host expression of post-translationally modified proteins in their native conformation (7) and, therefore, increase biological activity (23). Our previous study demonstrated that IL-12 DNA has a higher biological activity than IL-12 protein delivered by live recombinant *L. lactis*(26). Indeed, *L. lactis* is used to co-deliver protein and cDNA into mammalian cells (19). Using a single *L. lactis* vehicle system to co-deliver various therapeutic molecules via two distinct expression systems may eliminate the negative effects of simultaneous expression of multiple foreign genes. In addition, it may eliminate the conformational effects of the eukaryotic protein expressed. Previous studies (11,27) have shown the effective use of mucosal vaccination and immunotherapy against HPV-associated cervical cancer using mucosally co-administered live *L. lactis* strains expressing cell wall-anchored E7 and a secreted form of IL-12. These observations emphasize the advantages of i.n. routes of immunization over intragastric routes for inducing an antigen-specific immune response. In the present study, the differences in the antitumor effects of co-delivered E7 protein antigens and murine IL-12 DNA adjuvant by single or multiple recombinant lactococci were analyzed. Use of i.n. administration of recombinant *L. lactis-*carrying protein antigen and DNA adjuvant for eliciting an immune response was also assessed.

A strong E7-specific cellular immune response is the basis of the treatment and prevention of HPV-16-associated tumors. In the present study, E7 protein antigen and IL-12 DNA adjuvant delivered using one or two recombinant lactococci were able to induce strong CTL responses and subsequently enhance the tumor protection effect. Meanwhile, LL-IL-12_D_ and LL-E7_P_ alone exhibited only minor effects on CTL activities (Fig. 2). This was consistent with previous observations (11) and provides further support for the hypothesis that co-delivery of IL-12 and E7 may be useful for the induction of E7-specific CTL responses *in vivo*. The antitumor activity elicited by immunization with LL-E7_P_-IL-12_D_ and co-vaccination of LL-E7_P_ and LL-IL-12_D_ did not appear to be significantly different in terms of tumor size in tumor-bearing mice. However, the percentage of tumor-free mice and the survival rate of tumor-bearing mice challenged with TC-1 indicated that the antitumor effect of LL-E7_P_-IL-12_D_ was greater than that of LL-E7_P_/LL-IL-12_D_. Furthermore, the small difference in the tumor sizes of mice immunized with LL-E7_P_-IL-12_D_ compared with the other TC-1-challenged animal groups indicates that using a single *L. lactis* strain carrying recombinant protein antigen and DNA adjuvant may reduce individual differences in the immune response. In contrast to results of a previous study (11), the present study revealed that the LL-E7_P_-IL-12_D_ vaccine candidate induced only a limited degree of antibody response, which may be associated with low intracellular constitutive expression of E7 proteins in *L. lactis*(28) In addition, these effects may result from the direct delivery of E7 protein antigen into mammalian cells (18).

One noteworthy observation was that intranasally administered LL-IL-12_D_ has specific antitumor effects, which were different from the observations of Bermúdez-Humarán *et al*(11) in live *L. lactis* strains expressing a secreted form of IL-12. One possible explanation for this difference is that expression of IL-12 cDNA delivered by *L. lactis* may be sustained over a short period of time *in vivo*. For instance, serum IL-12 expression was observed until 5 days following i.n. administration of LL-IL-12_D_ in the present study (Fig. 5). Certain studies have indicated that systemic IL-12 therapies may be limited by high levels of toxicity (29). However, toxic effects were not observed in the present study, further demonstrating that IL-12 may be delivered safely and effectively via the i.n. route (30*)*.

The effective suppression of tumor growth in mice injected with TC-1 tumor cells demonstrated that the co-delivery of recombinant E7 protein antigen and IL-12 DNA adjuvant using a single *L. lactis* strain is able to effectively elicit an antigen-specific immune response. Additionally, i.n. administration of a single recombinant *L. lactis* strain carrying adjuvant and DNA antigen provides an effective alternative to traditional vaccination methods involving antigen production and purification barriers. The use of single recombinant lactococcal strains to co-deliver therapeutic proteins and DNA may also combine the advantages of protein vaccines with those of DNA vaccines.
